# Daily sleep quality and mood as predictors of pain in children with juvenile polyarticular arthritis

**DOI:** 10.1186/1546-0096-9-S1-P129

**Published:** 2011-09-14

**Authors:** MH Bromberg, KM Gil, LE Schanberg

**Affiliations:** 1 Department of Psychology, University of North Carolina, Chapel Hill, NC, USA; 2 Department of Pediatrics, Duke University Medical Center, Durham, NC, USA

## Background

Children with arthritis experience frequent pain, but the predictors of daily pain variations are largely unidentified. Sleep is a potential predictor of pain in children with arthritis.

## Aim

To examine sleep quality as a predictor of pain in children with arthritis and determine whether mood moderates the relationship. Based on theoretical models of pain and sleep in children, we also examined the alternate direction of influence, with pain serving as a predictor of sleep quality.

## Methods

In this prospective, longitudinal study, children with polyarticular arthritis (n = 51, ages 8-16 years, *m* = 12.4, *SD* = 2.8) tracked daily symptoms using paper diaries over 2 months. Children reported daily pain intensity and nightly sleep quality on visual analog scales. Daily mood was assessed via a standardized facial affective scale. Multilevel modeling, a statistical technique that allows the study of within-child (daily) and between-child (individual) sources of variance, was used for data analysis. Age and physician-rated disease severity were included as covariates.

## Results

Children completed an average of 50 diaries (*SD* = 13.67; 84% of days). Neither age nor disease severity predicted pain (*p* > .05). At the daily level, poorer sleep quality was associated with higher next-day pain ratings (*p* < .01)*.* Mood moderated this relationship such that as positive mood increased, the relationship between poor sleep quality and high pain weakened (*p* < .01). Daily pain did not predict nightly sleep quality (*p* > .05) but poorer sleep quality, predicted higher mean pain ratings (*p* < .05).

## Conclusions

Sleep quality is an important predictor of pain in children with arthritis. Results support the need for routine sleep assessment and interventions to improve sleep quality in children with arthritis. Interventions targeting mood may weaken the relationship between poor sleep quality and pain. These findings add to the growing body of literature on the utility of daily diaries for analyzing patterns of pain, sleep, and mood in children with chronic painful conditions.

